# Shedding Light on the Shade: How Nurseries Protect Their Children from Ultraviolet Radiation

**DOI:** 10.3390/ijerph15091793

**Published:** 2018-08-21

**Authors:** Cornelia Fiessler, Annette B. Pfahlberg, Wolfgang Uter, Olaf Gefeller

**Affiliations:** Department of Medical Informatics, Biometry and Epidemiology, Friedrich-Alexander University of Erlangen-Nuremberg, 91054 Erlangen, Germany; cornelia.fiessler@fau.de (C.F.); annette.pfahlberg@fau.de (A.B.P.); wolfgang.uter@fau.de (W.U.)

**Keywords:** ultraviolet radiation, shade, primary prevention, nursery, sun protection, children, skin cancer

## Abstract

Minimizing exposure to ultraviolet radiation (UVR) is strongly recommended as the most important primary prevention measure regarding skin cancer. The responsibility for adequate sun protection of young children lies with their parents and external caregivers. Since a high proportion of 3- to 6-year-old children in Germany attend nurseries, the practice of sun protection in this setting was assessed. A survey was conducted in 246 nurseries in southern Germany during spring and summer of 2014 and 2015. Shade coverage in the outdoor area of the nursery was assessed by study team members and UVR protective behavior of staff was assessed by an interview with the directors. On average, 52% of the entire outdoor area and 65% of the children’s outdoor play area were covered by shade, with a significant difference between nurseries of different sizes, pointing to a better shade coverage in larger nurseries. The daily outdoor stay was not regularly scheduled before or after peak sun intensity hours around noon to avoid intense UVR exposure. General sun protection rules were present in the majority of the nurseries and addressed predominantly wearing sunhats and applying sunscreen. Our findings show that current sun protection recommendations for children are only partially met in nurseries and indicate a lower level of sun protection in small institutions. Especially, avoidance of excessive exposure to UVR around noon and the importance of shade provision over play structures needs to be emphasized in future information campaigns.

## 1. Introduction

Rising incidence rates for skin cancer have been observed among almost all Caucasian populations worldwide over the last decades, making cutaneous malignant melanoma (CMM) and nonmelanoma skin cancer (NMSC) an important public health concern [1]. In Germany, the number of new diagnoses has increased fivefold since the 1970s, and 21,900 new cases are predicted for 2018 [2]. This development also leads to an increase in healthcare costs, since treatments of skin neoplasms have gone up [3,4].

Etiological research on skin cancer has shown that genetic predisposition, fair skin, a high number of melanocytic naevi and exposure to ultraviolet radiation (UVR) play a major role in the development of skin cancer, with exposure to UVR being the only exogenous and thereby modifiable risk factor [5,6,7]. UVR causes direct and indirect carcinogenic DNA damage of the skin, which ultimately induces skin cancer [8]. The most important prevention measure is therefore avoiding or reducing exposure to UVR. Accordingly, primary prevention campaigns have focused on communicating sun-protective behavior to enable individuals to take appropriate actions [9,10,11].

The amount of solar UVR received during lifetime is not uniformly distributed over all ages. Children experience substantially higher levels of UVR exposure through outdoor activities and recreation than adults [12]. At the same time, children have only a very limited knowledge of cause-and-effect relations regarding skin cancer [13]. They are thus unable to protect themselves adequately. Therefore, parents and external caregivers have a great responsibility to ensure sun protection measures are being applied to the children under their supervision.

A series of studies in Germany have hitherto focused on the parental perspective on children’s sun protection at home and on the beach [14,15,16,17,18,19,20]. Since a high proportion of 3- to 6-year-old children in Germany are supervised for several hours per day in nurseries, the caregivers at the nurseries play an important role for daily sun protection of children at this age [9]. Therefore, the current study aimed at evaluating the sun protection situation in nurseries more closely. In particular, we examined the shade coverage in the outdoor area of the nurseries, UVR protective behavior implemented by nursery staff, as well as the existence of sun protection guidelines or policies in nurseries and sources used by nursery directors to receive sun protection information.

## 2. Materials and Methods

### 2.1. Study Design

After a pilot phase with visits in two nurseries, the survey took place during the spring and summer months (i.e., mid-April until mid-September in the northern hemisphere) in 2014 and 2015. The study region was located between 49°22′ N and 49°55′ N in the northern part of Bavaria, a southern German federal state, and comprised of four cities (Erlangen, Fürth, Forchheim, Bamberg) and 83 rural municipalities in their immediate vicinity. The lowest solar zenith angle (SZA) corresponding to the highest altitude of the sun at the summer solstice equals 26° in the study region (differences in SZA within the study region due to varying locations are quite negligible and amount to a maximum of 0°25′). During the study period, higher SZA up to 56°25′ occurs in the study region (in mid-September). Most of the on-site visits took place between the beginning of May and the end of July, where the SZA in the study region ranges between 26° and 44°. The average UV index during spring and summer in the study region ranges between 4 (April, September) and 7 (June, July), but can occasionally attain a value of 9 under extreme weather conditions [21,22]. According to official administrative information, 343 nurseries existed in this region. Forest kindergartens (i.e., nurseries where children are supervised almost the whole time outdoors in a forest or other natural environment) and nurseries already visited during the pilot study were excluded. The remaining 336 nurseries were informed by letter about the study and afterwards contacted by phone to request participation. After consent to participate was achieved, an appointment for a personal interview including an on-site inspection of the outdoor area was made. The study has been approved by the local ethics committee at the University of Erlangen-Nuremberg.

### 2.2. Inspection of Shade in Outdoor Area

In the outdoor area of the nursery specifically trained members of the study team assessed the proportion covered by shade (i) for the overall area and (ii) restricted to ‘play areas’, i.e., subareas with play structures (such as swings or trampolines) or sandboxes. Instead of evaluating visible shade in the outdoor area, the study team members assessed shade coverage based on a theoretical standard scenario with a SZA fixed at 30° (taking into account wall shade from surrounding buildings in addition to shade provided by trees, garden parasols, shade sails, awnings and porches in the outdoor area of the nurseries). As a result, the assessment of shade coverage is independent of seasonal and diurnal effects as well as weather conditions at the day of inspection. To ensure a standardized assessment of shade coverage, training sessions for all study team members were performed. During the training sessions, the geometrical and physical fundamentals of estimating shade coverage were explained and practical exercises deepened the theoretical knowledge. During the first month of the field phase in both study years, nurseries were always visited jointly by two or three study team members who initially assessed shade coverage independently and subsequently determined a consensus result. These quality assurance activities aimed to increase agreement in shade assessment between different study team members and to avoid individual misunderstandings when implementing the procedure of estimating shade coverage. Furthermore, the different—not mutually exclusive—sources of shade in the outdoor area of the nurseries were determined (trees, garden parasols, shade sails, awnings, and porches).

### 2.3. Personal Interview

Representative for the nursery staff, the personal interview was conducted with the director of the nursery as the responsible person for the implementation of sun protection at the nursery. The questionnaire for the personal interview contained items regarding

(a) number and age range of supervised children,
(b) sun protection measures taken by the staff during outdoor playtime,
(c) requests to parents concerning sunscreen,
(d) existence of written policies and/or verbally communicated guidelines for the staff detailing sun-protective measures,
(e) the sources of information about sun protection used by the director.

The questions addressing (b) and (d) were open and answers were subsequently categorized by the interviewer into the following categories: ‘Seeking shade’, ‘avoiding peak sun intensity hours around noon’, ‘wearing long-sleeved clothes’, ‘wearing sunglasses’, ‘wearing sunhats’, and ‘applying sunscreen’. The question concerning (c) was also open and categorized into the following categories: ‘Applying sunscreen to children in the morning before handing them in the nursery’ and ‘providing sunscreen for their child for later use in the nursery’. For sources of information about sun protection, the directors were asked to indicate all of the following options that they had used: Information material from pharmacies or physicians, advertisements or posters, counselling at pharmacies or physicians, internet, magazine or newspaper articles, reports on TV, reports on the radio, talks at events, information from family/friends/neighbors, policies/guidelines, e.g., provided by the employer.

### 2.4. Statistical Analyses

Descriptive statistics (mean and standard deviation or absolute frequencies and percentages) were calculated to describe the distribution of answers from the interview, the shaded area and sources of shade (i) in the overall study sample and (ii) stratified by size of the nursery (small: <50 children, medium: 50–99 children, large: ≥100 children). Spearman’s rank correlation coefficient was calculated to evaluate the association between the proportion of the shaded area and the length of the outdoor stay. Differences between small, medium and large nurseries were analyzed using Jonckheere-Terpstra and Cochran-Armitage test, respectively. Both statistical tests are specifically designed to evaluate so-called trend alternatives, i.e., an ordered pattern of means and percentages, respectively, arranged monotonically from small to large nurseries. The Jonckheere-Terpstra test is a nonparametric approach not imposing a specific distributional assumption on the data. All statistical analyses were conducted using the statistical software package *R* (Version 3.3.1, *R* Core Team, Vienna, Austria) [23].

## 3. Results

### 3.1. Characteristics of Participating Nurseries

Of the 336 contacted nurseries, 247 (74%) agreed to participate in the study. One nursery could not be visited due to logistical reasons. The remaining 246 nurseries comprised of a total number of 2035 staff members and 15,643 supervised children, mostly between 2.5 and 6 years of age. Sixty-one (25%) of the nurseries were small (<50 attending children), 156 (63%) were medium-sized (50–99 children) and 29 (12%) were large (≥100 children). Regarding the type of sponsorship of the nurseries in our survey, most nurseries were run by churches or confessional welfare associations (*n* = 145, 59%), nearly a quarter was operated by the respective city or municipality (*n* = 59, 24%), a variety of private regional non-profit associations governed 19 (8%) nurseries, fifteen nurseries (6%) were run by national non-confessional welfare associations, four nurseries (2%) were operated by private non-profit companies, and the remaining four were run by parents’ initiatives (*n* = 3, 1%) and a private individual (*n* = 1). The interviewed directors were mostly female (97%) and on average 46.8 ± 9.7 years old.

### 3.2. Shade Coverage in Outdoor Area of Nurseries

Inspection of the outdoor area was possible in 243 of 246 nurseries. One nursery was renovated at the time of the study and two had only indoor areas and used nearby public playgrounds for their outdoor time instead.

**Figure 1:**
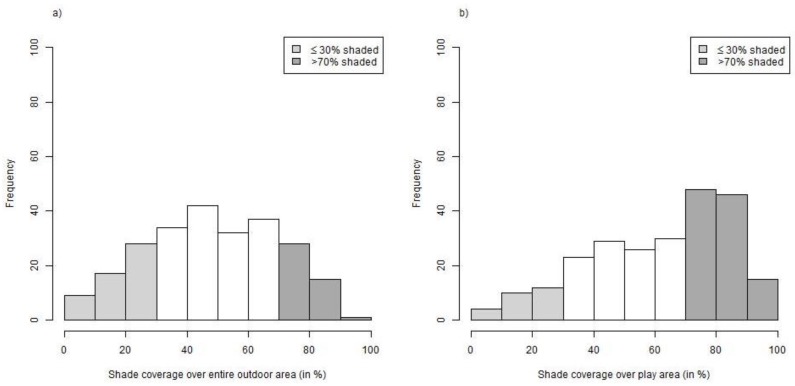
Histograms of shade coverage observed in 243 nurseries in Bavaria, southern Germany: (**a**) shade coverage over entire outdoor area; (**b**) shade coverage over play area.

The shade coverage in the entire outdoor areas of the nurseries showed a fairly symmetrical distribution (Figure 1a). On average, half of the entire outdoor area (52%) in the nurseries was shaded (Table 1). Fifty-four nurseries (22%) offered less than 30% of shade, while the outdoor areas of 44 nurseries (18%) had more than 70% of shade.

Shade coverage over areas with play structures (e.g., slides, swings or sandboxes) was higher, although still in 26 nurseries (11%) only 30% of this area was shaded (Figure 1b). Conversely, 45% of the nurseries provided more than 70% of shade over those areas. The overall mean percentage of shaded play areas was 65% (Table 1).

Almost all nurseries (99%) stated that their children spent time outside on all five days per week and on average 1.4 ± 0.9 h during peak sun intensity hours between 11 am and 3 pm (solar noon in the study region is attained around 1:15 pm during the study period). Twenty four percent of the interviewed nursery directors said that they would let the children spend more time outside if more shade was available. However, correlation between percentage of shaded area and length of daily outdoor stay was very low (0.01 and 0.05, respectively, for Spearman’s correlation coefficient between length of outdoor stay and shade coverage in the entire outdoor area and in the play area, respectively), strongly suggesting that the amount of shade is not considered when deciding how long the children can stay outside.

**Figure 2:**
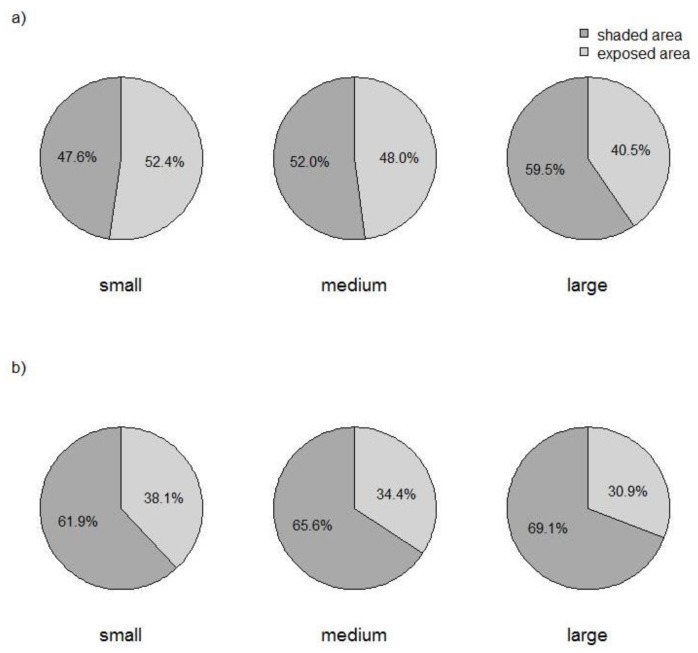
Mean proportion of shaded area observed in 243 nurseries in Bavaria, southern Germany, stratified by size of the nursery: (**a**) shade over entire outdoor area; (**b**) shade over play area. Small <50 children, medium 50–99 children, large ≥100 children.

In our study, the number of children attending the nursery was associated with shade coverage in the outdoor area. Larger nurseries offered comparatively more shade in their outdoor areas than small and medium-sized nurseries, revealing a significant trend (Table 1, Figure 2a,b). This finding applied to both the entire outdoor area as well as areas with play structures.

Trees as natural sources of shade existed in nearly all nurseries. Portable shade structures were present in 74% (garden parasols) and 76% (shade sails), while awnings and porches could be found in 33% and 64%, respectively, of the nurseries. These permanently installed shading systems were more frequent in larger nurseries.

### 3.3. Sun protection Behavior of Staff and Requests to Parents

Children were encouraged to seek shade while playing outside in 71% of the nurseries. In less than half of the nurseries (39%), the daily outdoor stay was scheduled before or after the peak sun intensity hours around noon (Table 2).

Long-sleeved clothes and sunglasses played a minor role in the daily sun protection routine in nurseries, probably due to the fact that the parents’ cooperation in supplying appropriate equipment is essential here. On the other hand, 85% of the nurseries required the parents to deliver their children in the morning already protected by sunscreen and 65% asked the parents to provide their children with their own sunscreen for possible re-application in the nursery.

Sun protection measures most commonly implemented were still applying sunscreen and wearing sunhats (99% and 98% of the nurseries, respectively). Additional alternative sunhats were available in 78% of the nurseries for children who forgot their own hat.

Regarding the size of the nursery, a clear trend could also be observed here, since larger nurseries implemented more often important UVR preventing measures like seeking shade or avoiding peak sun intensity hours than smaller nurseries.

### 3.4. Sun Protection Policies and Guidelines in Nurseries

Overall, the majority of directors (86%) confirmed that general sun protection rules, which applied to all employees, exist in their nursery. In 68% of the nurseries, those rules were verbally communicated to the staff, while in 18% a written sun protection policy was available. Large nurseries used written policies more often than small nurseries (Table 3).

Similar to the sun protection measures taken by the nursery staff, wearing sunhats and applying sunscreen were the topics most frequently covered in guidelines and policies. UVR preventing measures like seeking shade and avoiding peak sun intensity hours were only present in 7% and 5%, respectively, of the written sun protection policies, and were verbally communicated in 31% and 19%, respectively, of the nurseries. Guidelines or policies concerning long-sleeved clothing were rare and sunglasses were never mentioned in this context by the directors.

### 3.5. Sources of Information about Sun Protection

Table 4 shows the sources of information about sun protection mentioned by the directors. Most nursery directors stated that they used printed materials like brochures from pharmacies or physicians (88%) and magazine or newspaper articles (85%) for information about sun protection. Less than half of the participants were directly informed by pharmacies or physicians (42%) and family, friends or neighbors (49%). Only one of four directors (27%) used the internet as a source of information about sun protection behavior. No relevant differences in answers were apparent between directors from nurseries of different sizes.

## 4. Discussion

The high level of UVR exposure received during childhood has brought the topic of parental sun protection behavior into the focus of primary prevention campaigns like the SunSmart program in Australia or the project “Clever in Sonne und Schatten” from the German Cancer Aid [11,24], but research about the sun protection situation in daycare institutions is still scarce, especially outside Australia. Our large survey among 246 nursery directors, including an objective on-site assessment of shade coverage in the outdoor areas of the nurseries, gives new insights into the status quo of children’s sun protection in German daycare institutions and points to some deficits regarding the shade coverage in the nurseries’ outdoor areas and sun protection measures, especially in small nurseries.

A sufficient shade infrastructure over areas with play structures like sandboxes or slides is indispensable, since children tend to spend longer time in these areas when playing outdoors. In the nurseries from our survey, only two-thirds of those areas were shaded on average, which unnecessarily exposes the children to hazardous UV radiation in the remaining exposed areas. This result illustrates the need for better shade equipment in all nurseries, but particularly in small nurseries, where outdoor play areas were shaded to a significantly lesser extent.

Scheduling outdoor time either before or after peak sun intensity hours was mentioned in our study by less than half of all nursery directors, which is similar to other studies [25,26]. One possible reason for this disappointing result is that the positive effects of staying outdoors on children’s health, e.g., increased physical activity or sufficient vitamin D levels, are rated higher than potential harmful consequences due to UVR exposure. Generally, children attending the nurseries spend time outside every day, even including more than one hour during peak UVR hours around noon on average, which is in conflict with recommendations for sun protection [27].

Most directors in our study stated that they encourage the children to seek shade while playing outside. Results on shade seeking behavior in earlier nursery studies are diverse, ranging from a high proportion of nurseries paying attention to this aspect [25,28,29,30,31] to a sporadic and infrequent protection by shade [32]. Some studies did not address this part of sun-protective behavior at all [26,33]. Today, the World Health Organization (WHO) and other national health organizations strongly recommend seeking shade as one of the primary skin cancer prevention measures [27,34]. Our finding shows that awareness for this sun protection component may have risen, but should still be emphasized in future skin cancer prevention campaigns due to its relevance.

However, although the general awareness among nursery directors for UVR preventing measures like seeking shade and avoiding peak sun intensity hours is encouraging, participants of small nurseries especially mentioned these important sun protection practices considerably less often than medium-sized or large nurseries’ directors. Whether this is due to a lack of knowledge, as previous information campaigns may have focused on larger nurseries where more caregivers and parents could be reached, or due to the lower shade coverage in small nurseries, cannot be determined. Nevertheless, the lower awareness for this UVR preventing behavior combined with lesser shade coverage highlights the necessity to inform also small institutions sufficiently.

The sun protection practices mentioned least included wearing long-sleeved clothing and sunglasses. Long-sleeved clothes act as a textile shield to UVR and—in contrast to sunscreen—they provide water and sweat resistant sun protection [35,36]. A new index, the Garment Protection Factor, joins the two important aspects for determining the extent of sun protection offered by clothing, namely the quality of UVR blocking offered by the fabric and the coverage level of the body surface area due to clothing design, and informs the public about the sun-protective properties of clothing [37,38]. The difficulty for nurseries lies, however, in the fact that parents have to provide the necessary items, and as several studies have shown, parents themselves are often not aware of the importance of these two sun protection practices [14,39]. The fact that the majority of nurseries included the parents in the daily sun protection routine (by requesting to deliver the children with sunscreen applied or providing sunscreen) potentially indicates an increased awareness of the directors for the importance of a comprehensive sun protection strategy. A future study evaluating options for joint information or intervention campaigns for both parents and external caregivers is recommended.

Wearing sunhats and applying sunscreen was common among nurseries in our study. Earlier studies have partially found obstacles regarding the application of sunscreen in nurseries because parents were concerned about allergic reactions to certain products [9]. Our survey revealed that nurseries have meanwhile found a solution suitable for daily use by asking the parents to provide their children with their own well-tolerated sunscreen product. However, the effectiveness of sunscreen in general still depends strongly on the right application regarding amount and regular re-application. It should therefore only be used in combination with other, more prioritized sun-protective practices. The very high percentage of nursery directors mentioning sunscreen use indicates a possible over-reliance on this practice, which should be addressed in future information campaigns.

Previous studies have found a positive effect of skin cancer prevention programs for childcare centers on the attitudes, knowledge and behavior of the staff [9,25,32]. The long-term impact of such campaigns is still unclear and due to a high staff turnover in childcare institutions, repeated interventions are suggested. However, as a result of increased pedagogical and administrative demands, many nurseries—especially small institutions—do not have enough staff to participate regularly in these programs. Alternatively, a written sun protection policy listing the recommended measures may secure the implementation of a reliable sun protection standard in nurseries. In our study, written policies were more frequent in large nurseries, which might at least partially explain the greater awareness of their directors for important sun protection practices like seeking shade and avoiding outdoor stay around noon.

The utilization of the internet for healthcare applications in general is increasing. Not only is it used to inform the population about health topics, but also to promote health-conscious behavior [40,41]. The Australian SunSmart Campaign has recently released a smartphone app which inter alia informs about UVR levels and sends personalized sun protection messages [42]. Our study shows that the internet is infrequently consulted by nursery directors for information seeking about sun protection, which is similar to findings by other studies assessing the information channels used for acquiring knowledge on sun protection [43]. The participants in our survey used mainly conventional media like information brochures or magazine articles, which impedes web-based sun protection campaigns for this target group. Future research is necessary to determine to what extent more personalized channels like social media and apps can contribute to the efficacy of health prevention campaigns.

Our study had also some limitations. Even though the response was high, we cannot determine if the sample was representative for all childcare institutions in this region or even in Germany. Although the type of sponsorship of the nurseries showed no association with shade coverage and sun-protective measures, there might have been some impact of the operating association of the nurseries on these issues, e.g., due to unequal levels of funding or institutional guidelines, that could not be determined in our study because of insufficient information. Given such a relationship existed, we cannot rule out that it might have confounded the analysis of the influence of nursery size on shade provision and sun-protective measures. Assessment of shade coverage in our study was based on a theoretical scenario with a SZA fixed at 30°, which leads to a slight overestimation of the level of actual shade around solar noon during the weeks near to the summer solstice. The advantage of obtaining comparable assessments of shade coverage independent of weather conditions and seasonal as well as diurnal effects led us to adopt ascertaining shade coverage using this theoretical standardized scenario instead of alternative methods. The aspect of differences in the extent of UVR protection offered by the shade resulting from different transmissive materials of shade sails or garden parasols and varying levels of diffuse UV radiation due to different ground and surrounding conditions in the outdoor area of the nurseries has not been addressed in our study [44]. These aspects should be included in future studies. Besides, it was not possible to interview every single staff member in the nurseries; thus, we had to rely on the answers given by the director. Nursery directors may have answered the questions about their sun-protective practices in a way that was perceived as socially desirable. However, this effect was likely mitigated by using open questions, since it is more difficult to choose potentially desirable answers if no options are given, compared to selecting from a set of possible pre-defined answers.

## 5. Conclusions

Our study among 246 nursery directors revealed a high awareness of common sun protection measures such as wearing sunhats and using sunscreen, but also identified shortcomings regarding avoidance of peak sun intensity hours, use of long-sleeved clothes and provision of shade over areas with play structures where children stay longer. Future campaigns should emphasize these important components of sun protection and ensure that small nurseries are especially well-informed about the prevention of UVR exposure and the benefits of an adequate shade infrastructure. Template policy documents made available to the nurseries may aid in achieving a higher, and more standardized, level of UVR protection. The efficiency of such policies in German nurseries should be the subject of further research.

## Figures and Tables

**Table 1:** Results from the on-site inspection of outdoor areas in 243 nurseries in Bavaria, southern Germany. Numbers represent mean values and standard deviation (SD) for the proportion of area covered by shade, and absolute numbers and percentages for the sources of shade.

Shade Coverage and Sources	Overall	Small ^3^	Medium ^3^	Large ^3^	*p*
*n*	243	59	155	29	
Shade coverage in entire outdoor area (in %, mean (SD))	51.81 (21.45)	47.63 (23.03)	51.97 (20.68)	59.48 (20.72)	0.011 ^1^
Shade coverage in play area (in %, mean (SD))	65.11 (22.88)	61.86 (22.91)	65.59 (22.25)	69.10 (25.94)	0.033 ^1^
Trees (*n* (%))	235 (96.7)	56 (94.9)	152 (98.1)	27 (93.1)	0.994 ^2^
Garden parasols (*n* (%))	180 (74.1)	43 (72.9)	114 (73.5)	23 (79.3)	0.582 ^2^
Shade sails (*n* (%))	185 (76.1)	43 (72.9)	122 (78.7)	20 (69.0)	0.967 ^2^
Awnings (*n* (%))	81 (33.3)	15 (25.4)	55 (35.5)	11 (37.9)	0.167 ^2^
Porches (*n* (%))	157 (64.6)	35 (59.3)	100 (64.5)	22 (75.9)	0.147 ^2^

^1^ Jonckheere-Terpstra Test; ^2^ Cochran-Armitage Test; ^3^ small <50 children, medium 50–99 children, large ≥100 children.

**Table 2:** Sun protection measures taken by the nursery staff during outdoor playtime and requests to parents concerning sunscreen in 246 nurseries in Bavaria, southern Germany.

Sun Protection Measures	Overall	Small ^3^	Medium ^3^	Large ^3^	*p*
*n*	246	61	156	29	
Sun protection measures taken by staff:					
Seeking shade (*n* (%))	175 (71.1)	39 (63.9)	113 (72.4)	23 (79.3)	0.108 ^1^
Avoiding peak sun intensity hours (*n* (%))	96 (39.0)	23 (37.7)	59 (37.8)	14 (48.3)	0.441 ^1^
Wearing long-sleeved clothing (*n* (%))	29 (11.8)	5 (8.2)	17 (10.9)	7 (24.1)	0.054 ^1^
Wearing sunglasses (*n* (%))	6 (2.4)	2 (3.3)	2 (1.3)	2 (6.9)	0.586 ^1^
Wearing sunhats (*n* (%))	242 (98.4)	59 (96.7)	154 (98.7)	29 (100.0)	n.a. ^2^
Applying sunscreen (*n* (%))	243 (98.8)	61 (100.0)	156 (100.0)	26 (89.7)	n.a. ^2^
Additional sunhats available	191 (77.6)	50 (82.0)	119 (76.3)	22 (75.9)	0.415 ^1^
Requests to parents concerning sunscreen:					
Deliver children with sunscreen already applied (*n* (%))	209 (85.0)	48 (78.7)	136 (87.2)	25 (86.2)	0.207 ^1^
Provide sunscreen for their child for later use in nursery (*n* (%))	161 (65.4)	36 (59.0)	106 (67.9)	19 (65.5)	0.372 ^1^

^1^ Cochran-Armitage test; ^2^ *p*-values were not available (n.a.) due to zero counts in contingency table; ^3^ small <50 children, medium 50–99 children, large ≥100 children.

**Table 3:** Written policies and verbally communicated guidelines regarding sun protection existent in 245 nurseries in Bavaria, southern Germany.

Policies and Guidelines	Overall	Small ^3^	Medium ^3^	Large ^3^	*p*
*n*	245	60	156	29	
Written policies:					
Seeking shade (*n* (%))	17 (6.9)	3 (5.0)	11 (7.1)	3 (10.3)	0.360 ^1^
Avoiding peak sun intensity hours (*n* (%))	12 (4.9)	1 (1.7)	9 (5.8)	2 (6.9)	0.207 ^1^
Long-sleeved clothing (*n* (%))	1 (0.4)	0 (0.0)	0 (0.0)	1 (3.4)	n.a. ^2^
Sunhats (*n* (%))	36 (14.7)	7 (11.7)	22 (14.1)	7 (24.1)	0.164 ^1^
Sunscreen (*n* (%))	35 (14.3)	8 (13.3)	20 (12.8)	7 (24.1)	0.289 ^1^
Verbally communicated guidelines:					
Seeking shade (*n* (%))	75 (30.6)	20 (33.3)	48 (30.8)	7 (24.1)	0.410 ^1^
Avoiding peak sun intensity hours (*n* (%))	46 (18.8)	9 (15.0)	32 (20.5)	5 (17.2)	0.614 ^1^
Long-sleeved clothing (*n* (%))	4 (1.6)	1 (1.7)	3 (1.9)	0 (0.0)	n.a. ^2^
Sunhats (*n* (%))	135 (55.1)	34 (56.7)	84 (53.8)	17 (58.6)	0.986 ^1^
Sunscreen (*n* (%))	138 (56.3)	37 (61.7)	85 (54.5)	16 (55.2)	0.440 ^1^

^1^ Cochran-Armitage test; ^2^ *p*-values were not available (n.a.) due to zero counts in the contingency table; ^3^ small <50 children, medium 50–99 children, large ≥100 children.

**Table 4:** Sources of information about sun protection used by directors of 246 nurseries in Bavaria, southern Germany.

Sources of Information about Sun Protection	Overall	Small ^2^	Medium ^2^	Large ^2^	*p*
*n*	246	61	156	29	
Information material from pharmacies or physicians (*n* (%))	217 (88.2)	50 (82.0)	140 (89.7)	27 (93.1)	0.081 ^1^
Advertisements or posters (*n* (%))	167 (67.9)	40 (65.6)	110 (70.5)	17 (58.6)	0.768 ^1^
Counseling at pharmacies or physicians (*n* (%))	104 (42.3)	27 (44.3)	61 (39.1)	16 (55.2)	0.581 ^1^
Internet (*n* (%))	67 (27.2)	14 (23.0)	47 (30.1)	6 (20.7)	0.863 ^1^
Magazine or newspaper articles (*n* (%))	208 (84.6)	53 (86.9)	127 (81.4)	28 (96.6)	0.540 ^1^
Reports on TV (*n* (%))	178 (72.4)	44 (72.1)	116 (74.4)	18 (62.1)	0.493 ^1^
Reports on the radio (*n* (%))	101 (41.1)	23 (37.7)	63 (40.4)	15 (51.7)	0.261 ^1^
Talks at events (*n* (%))	36 (14.6)	9 (14.8)	26 (16.7)	1 (3.4)	0.312 ^1^
Information from family, friends, neighbors (*n* (%))	121 (49.2)	30 (49.2)	76 (48.7)	15 (51.7)	0.873 ^1^
Policies or guidelines (*n* (%))	110 (44.7)	26 (42.6)	68 (43.6)	16 (55.2)	0.351 ^1^

^1^ Cochran-Armitage test; ^2^ small <50 children, medium 50–99 children, large ≥100 children.
